# Editorial: the political determinants of health inequities and universal health coverage

**DOI:** 10.1186/s12992-019-0514-6

**Published:** 2019-11-28

**Authors:** Sonja Kristine Kittelsen, Sakiko Fukuda-Parr, Katerini Tagmatarchi Storeng

**Affiliations:** 1 Centre for Development and the Environment (SUM), University of Oslo, Oslo, Norway; 2 Julien J. Studley Graduate Programs in International Affairs, The New School, New York City, NY USA; 3 Department of Epidemiology and Population Health, London School of Hygiene and Tropical Medicine, London, UK

## Background

Universal health coverage (UHC) is “a political choice”, declared the World Health Organization’s Director-General at the High-Level Meeting on UHC on September 23, 2019 [1]. The meeting brought together heads of state, political leaders, health advocates and policymakers to renew financial and political commitment to achieving UHC by 2030, in keeping with target 3.8 of the Sustainable Development Goals (SDGs). But what precisely does it mean to state that UHC is political?

According to the WHO, UHC means that, “all people and communities can use the…health services they need, of sufficient quality to be effective, while also ensuring that the use of these services does not expose the user to financial hardship” [2]. Yet, what precisely UHC entails and the type of society envisioned by it remains highly contested, often revealing competing conceptualizations of health as a commodity versus health as a right [3, 4]. Critics have argued, for example, that the current promotion of UHC is “silent on social determinants of health and community participation” and underplays how expanding insurance coverage opens up healthcare to private sector capture and the further medicalization and commercialization of health [5]. There are mounting concerns that current directions in UHC policy choices – notably, the privatization of healthcare and the shrinking role of the state in the stewardship and provision of health services – have the potential to widen inequalities [3, 5, 6].

This special issue of *Globalization & Health* is motivated by a commitment to addressing such concerns analytically and empirically by studying “the power and politics behind choices to expand healthcare access” [7] or how policies *outside* of the health sector shape moves towards UHC. To do so, the issue brings together critical historical and social scientific analyses of the power relations and political processes that shape decision-making on UHC. These contributions were first presented and discussed at a conference on the political origins of health inequity and universal health coverage convened by the Independent Panel on Global Governance for Health in November 2018 in Oslo, Norway.

The Independent Panel was established in 2016 to follow up on the work of the Lancet-University of Oslo Commission on Global Governance for Health. In 2014, the Commission published a report that drew attention to the *political* determinants of health inequities – that is, “the transnational norms, policies and practices that arise from political interaction across all sectors that affect health” [8]. The Commission report argued that while states have a key role in ensuring their populations’ right to health, many determinants of health are now beyond the control of single governments; rather, with globalization, the inequities in health that exist both within and between countries are increasingly determined by transnational activities and global political interaction involving actors with different interests and degrees of power. In order to address health inequities, the Commission argued, it is necessary to strengthen the global governance system, including addressing the global power asymmetries and powerful interests that are resistant to change, and to do so across all sectors that affect health.

The Independent Panel’s mandate is to take the Commission’s agenda forward, for example by challenging the silo thinking that constrains efforts to address health as an issue in the global institutions beyond the health sector. To this end, the 2018 Oslo conference brought together researchers, policymakers and activists from around the world to cross the divides between sectors (health vs. trade, environment, etc.), between functions (researchers vs. practitioners) and between academic disciplines (e.g. political science vs. anthropology). We selected UHC as a focus for the conference precisely because it is a policy area that brings governments face to face with political dilemmas relating to global governance challenges. For example, the provisions regarding intellectual property in trade and investment agreements have major consequences in restricting the policy space of countries in designing equitable public health policies [9–11]. The contributions included in this special issue provide new perspectives on how asymmetries in power shape UHC, how UHC reforms intersect with broader national political struggles and histories, and how trade, financialized capitalism, and private interests shape UHC approaches globally.

### Power asymmetries as a political determinant of health inequity

A central argument advanced by The Lancet-University of Oslo Commission report is that power asymmetries feature prominently in the global governance landscape and are a key determinant of health inequity, limiting the range of choice and constraining action [8]. Yet, while power is central to governance processes and health equity debates, how power manifests in practice in global governance for health remains poorly understood [12]. Indeed, there have been recent calls for “a more rigorous analysis of how national, international, and institutional actors shape and influence the global political determinants of health” [12].

The articles by Suerie Moon and Alexander Kentikelenis and Connor Rochford in this special issue respond to this call by elaborating conceptual frameworks for understanding power dynamics. Moon defines power in global governance “as the ability to shape the thinking and/or actions of other actors in the global public domain”. Taking her starting point in the recognition of global health as a complex adaptive system, Moon argues that the widely used and established conceptualizations of power in the social sciences literature – Barnett and Duvall, Dahl, Bourdieu – are too narrow and simple to provide a useful framework for analysis of global health. She then provides a typology of eight kinds of power – physical, economic, structural, institutional, moral, discursive, expert, and network-based – as a means of identifying how different actors wield power in global governance, and how these different types of power interact. Given that complex adaptive systems involve multiple agents interacting in multiple ways, and are often characterized by “butterfly” effects in which a small action in one part of the system can carry ramifications elsewhere, this implies that both different types of power and actors traditionally considered weak can exert influence on governance outcomes. Moon’s typology thus aims to move beyond a concept of power limited to “‘significant’ instances of influence” to also account for how seemingly smaller actions can carry weight, concluding that while power asymmetries are enormous, they are “neither absolute nor immutable”.

Kentikelenis and Rochford, in turn, propose a framework encompassing individual agency and institutional structures to account for how different types of power – political, economic, symbolic, and epistemic – manifest and interact across three levels of analysis: macro (e.g. neoliberalism as a dominant institutional order), meso (organizations operating within this institutional environment), and micro (individuals). They apply this framework to describe how neoliberalism impacts health equity through distinct “pathways of influence”. These include, for example, how dominant neoliberal frames manifest in the ideas, values and choices of individuals, such as an individual’s turn to medical crowdfunding, or how the increased prevalence of private actors in health impacts policy choices and the space available to address inequity.

These different forms of power identified provide a conceptual starting point for interrogating how power manifests in global governance to inform how UHC is defined and pursued, which are picked up and illustrated in a number of the other contributions to this special issue. Taken together, these papers contribute to both conceptual and empirical clarification on the function of power in the governance of health and in determining health outcomes.

### Trade, financialized capitalism, and the rise of private actors in healthcare

A recent editorial in *Review of International Political Economy* calls for further critical engagement on how capitalism as a global phenomenon interacts with health, since “The deeper structural foundations of health governance and the basic drivers of global health outcomes are often obscured” in literature examining the political dynamics of health [13]. Susan Sell takes up this challenge in her contribution to this special issue, by arguing that the structures of twenty-first century capitalism - characterized by financialization, trade driven by intangibles and global value chains, and associated rising inequality – pose fundamental challenges to the achievement of UHC. She points to multiple pathways where these structures undermine governments’ ability to fund programs from their domestic tax bases – capital mobility facilitates tax evasion, declining trade in goods leads to shrinking tax base in wages and resort to regressive taxes and crowdsourcing. She also points to important effects on the distribution of power and policy influence – the rising power of corporations in the intangibles sector driving monopoly capitalism, the declining role of labor unions, and countries negotiating trade agreements in favor of capital (strong intellectual property and capital liberalization) rather than markets for their workers. At individual level, there has been a shift in obligation from public provision of health services to an individualized responsibility for health outcomes where health is increasingly commodified and the citizen is recast as consumer. Sell identifies financial stability and adequate tax revenue as to important prerequisites for achieving UHC.

The pharmaceutical sector is one of the most significant cases of the intangible sector. The public health effects of intellectual property provisions of the WTO TRIPS agreement are well known. The achievement of UHC in the twenty-first century faces a stiffer challenge with the advent of bilateral and other plurilateral agreements that are broader and deeper in scope, extending beyond trade to investment matters that have the potential to carry significant consequences for countries’ policy space for UHC. As Deborah Gleeson and her coauthors point out, “there is no doubt that future trade agreements will continue to present a wide range of potential intersections with pharmaceutical policy”. Gleeson and colleagues provide an analytical framework for examining the ways in which twenty-first century trade and investment agreements impact pharmaceutical policy, focusing on four core objectives that are important for the pursuit of UHC: access and affordability; safety, efficacy and quality; rational use of medicines; and local production capacity and health security. While there is a large literature on the effects of intellectual property, this paper looks at the broader provisions of the post-WTO plurilateral agreements. They include the strengthened intellectual protection provisions (‘TRIPS plus’) but also extend to issues such as the regulation of pharmaceutical marketing/pricing/testing, investment protection, government procurement, sate-owned enterprises, customs administration, and regulatory coherence.

Nora Kenworthy provides a rich ethnographic example of how the changes in global capitalism discussed in Sell’s paper has facilitated the displacement of the state as a provider of health services in favor of a culture of individualized responsibility and commodified healthcare in the United States and also, increasingly, globally. Kenworthy’s article focuses on the global rise of medical crowdfunding platforms – a phenomenon she argues is fueled by gaps in the social safety net, rising healthcare costs and inadequate access to health services. She demonstrates how crowdfunding impacts health equity and health politics as both a technological, commercial and political determinant of health. Crowdfunding platforms, Kenworthy maintains, through their design, algorithms and data ownership practices, exacerbate inequities by mediating access to key health services based on an individual’s ability to successfully navigate the platform and compete against others to raise funds for their healthcare. As many of these platforms, like GoFundMe, are either owned by, or have ties to the corporate world, pertinent questions arise as to the ownership, use and access of the often sensitive health data provided by individuals in their crowdfunding campaigns. Kenworthy argues that crowdfunding is a deeply regressive public health strategy that is poorly suited for addressing the determinants of health or providing preventative services, since priority-setting is relegated to the haphazard pattern of individually designed crowdfunding campaigns, with acute healthcare needs often attracting the most funds. Kenworthy shows the importance of scrutinizing how medical crowdfunding platforms alter public values and discourses as to the rights and entitlements of citizens to health, including how these platforms displace a vision of health justice derived from a social contract with the state to one rooted in an individualized and marketized approach to healthcare.

James Pfeiffer and Rachel Chapman comment on how years of austerity and conditionalities under the International Monetary Fund in Mozambique have undermined public healthcare and encouraged an influx of international non-governmental organizations (NGOs), constraining the state’s ability to pursue UHC. They demonstrate how the policies and practices of international financial and donor institutions have channeled financial resources and personnel away from the public sector to NGOs, contractors and for-profit companies, setting up parallel systems that are fundamentally at odds with UHC.

Together, these contributions demonstrate how powerful market forces and actors outside of the health sector influence the norms, practices and policies of healthcare, and how power exercised through twenty-first century capitalism, technology and corporate interest manifests vis-à-vis individuals and the state to shape access to, and the provision of healthcare. They illustrate how fundamental shifts in the global political economy, characterized by processes of liberalization and financialization, and an increased presence of private actors in the provisioning and financing of healthcare, set constraints and shape expectations on how UHC is pursued.

### National struggles for health equity and universal health coverage: political drivers

Global policy discourse pits UHC as a universalist, utopian vision. But what does it mean to speak of ‘universal’, ‘health’ and ‘coverage’ in contemporary UHC policy and discourse? As Anne-Emanuelle Birn and Laura Nervi explain in their paper, despite its roots in 19th and twentieth century struggles for social rights, public health systems and welfare states, the contemporary model of UHC promoted by prominent global actors and articulated in the SDGs is reductionist in its vision, with inadequate attention to what ‘health’ entails beyond simply healthcare access. As a consequence, the social and societal aspects of health are sidestepped and health is rendered a site of individualized, biomedical, technical and depoliticized intervention. Drawing examples from Latin America, they argue for the need to take back the terms of debate, recognizing that the struggle for health justice is rooted in broader social and economic struggles for equity at both national and international levels.

Indeed, national histories and interactions between international and domestic forces and actors are central to understanding struggles for UHC in specific national contexts, as the contributions from Sri Lanka, India and Japan show. In her paper, Ramya Kumar traces the shifts from the publicly financed and delivered healthcare system established under the 1951 ‘Free Health’ policy to the current model of UHC outlined in the 2018 reforms that advocate for greater public-private partnership, including the public financing of private healthcare delivery. She argues that the current push towards further private sector collaboration risks reducing the fiscal space available to expand and improve public healthcare, and weakening the public sector by diverting public funds and human resources to the private sector.

In Brazil, meanwhile, political struggles have surrounded the pursuit of UHC from the mid-1980s, when the Unified Health System (SUS) was introduced, until present day. Cristiani Vieira Machado and Gulnar Azevedo e Silva describe the interplay of international and national political factors in the initial establishment of the SUS and in the challenges of implementing a universal health system in the years that have followed. In trends similar to those seen in Sri Lanka, insufficient public funding and the changing nature of public-private cooperation in healthcare have posed challenges in implementing a universal public health system and in reducing health inequalities in Brazil. While SUS has led to some important health gains, sustaining and furthering these gains hinge upon the future of democracy in Brazil, the continued political support for a public and universal health system, and on a strengthened ability of the state to regulate the private sector.

The important role of both government funding and state regulation is highlighted in the experience of Japan, where UHC is publicly funded while providers are in both public and private sectors. Naoki Ikegami argues that central to achieving health equity in Japan has been price regulation, known as the ‘fee schedule’ that sets prices for medicines, doctors’ fees, and all services, so that costs to patients (insurance premiums, copayments) are kept at affordable levels, and pressures on government budgets are contained. The policy also allows government to allocate resources in the public interest, such as by prioritizing primary care over specialized treatment by managing annual revisions to the fee schedule. Policy management is not just technocratic but shaped by negotiations with the providers, and the emphasis on primary health is no doubt related to the dominant role of primary health physicians in the powerful Japanese Medical Association. Looking forward, the system that was developed in the context of a growing economy is threatened by acute fiscal challenges posed by an aging demographic and a stagnant economy.

These country studies illustrate the different power hierarchies in specific national contexts. But they also illustrate a common theme: the fundamentally new challenges posed by the global environment of twenty-first century financialized capitalism, and rising inequality of income and wealth within and between countries.

## Conclusion

This special issue highlights the fundamentally political nature of UHC, illustrating the complex interaction between actors and processes at international and domestic levels in determining what UHC is and how it should be pursued. The contributions describe the political struggles between competing ideologies as to what UHC entails, including those over the role of the state vis-à-vis the private sector in the financing and delivery of healthcare against shifting economic and political contexts. These competing visions and approaches to UHC raise questions as to the kind of power being exercised in setting the agenda for UHC in any given context, as Moon and Kentikelenis and Rochford alert us to. This special issue, however, also raises the possibility of alternative approaches to the pursuit of UHC – approaches that envision a stronger role for the state in the stewardship of the health system and rooted in broader political struggles for social, economic and health justice.

These analyses point to important priorities for further research on the politics of UHC, notably more in-depth empirical studies of political processes that would help identify key obstacles to health equity within national and global governance structures. More importantly, a better understanding of the politics – the methods, ideas and alliances – that succeed in developing policy approaches that promote equity would contribute to theory building and policy advocacy.

## Data Availability

Not applicable.
